# Taft's Practical Treatise on Operative Dentistry

**Published:** 1883-01

**Authors:** 


### BIBLIOGRAPHICAL.

---

*A Practical Treatise on Operative Dentistry.*—By J. Taft. M. D., D. D. S., Professor of Principles and Pratice of Operative Dentistry in the Dental College of the University of Michigan. Fourth Edition. Revised, with one hundred and thirty six illustrations, Publishers: P. Blackiston, Son & Co. Philadelphia.

We gladly welcome another edition of this standard work; and, although the author announces that the changes in the new edition have not been so great as those in the former edition yet we find while a number of instruments and appliances have been omitted as superseded, others of recent invention have been added.

We have from time to time reviewed the different editions of this standard work which is so favorably known and appreciated as to require no further praise at our hands. The style and composition of the new edition are in no respect inferior to those of other years, and are creditable to the well known Publishers.

